# Thrombopoietin receptor agonists in inherited thrombocytopenias: an evolving mosaic of evidence

**DOI:** 10.1016/j.rpth.2026.106907

**Published:** 2026-08-12

**Authors:** Carlo Zaninetti

**Affiliations:** Department of Transfusion Medicine, University Medicine Greifswald, Greifswald, Germany

Thrombopoietin receptor agonists (TPO-RAs) represent a major therapeutic advance in inherited thrombocytopenias in recent years [1]. The orally available, non-peptide small molecule eltrombopag is the most extensively studied agent in this field. Small clinical trials and case series have shown its ability to transiently increase platelet counts and reduce bleeding in a subgroup of inherited thrombocytopenias, including the most prevalent forms worldwide: *MYH9*-related disease (*MYH9*-RD), monoallelic Bernard–Soulier syndrome, *ANKRD26-*related thrombocytopenia (*ANKRD26*-RT), and Wiskott–Aldrich syndrome [[2], [3], [4], [5]]. Based on these data, eltrombopag has been used as preoperative treatment in *MYH9*-RD, with 17 procedures reported to date, and more sporadically in other conditions [[6], [7], [8], [9], [10], [11], [12], [13], [14], [15]]. This use remains off-label, as no TPO-RA is currently approved for inherited thrombocytopenias. In most reported cases, treatment allowed surgery without prophylactic or therapeutic platelet transfusion, with no reported safety concerns. The key milestones in the clinical use of eltrombopag for inherited thrombocytopenias are summarized in the Figure.

**Figure:**
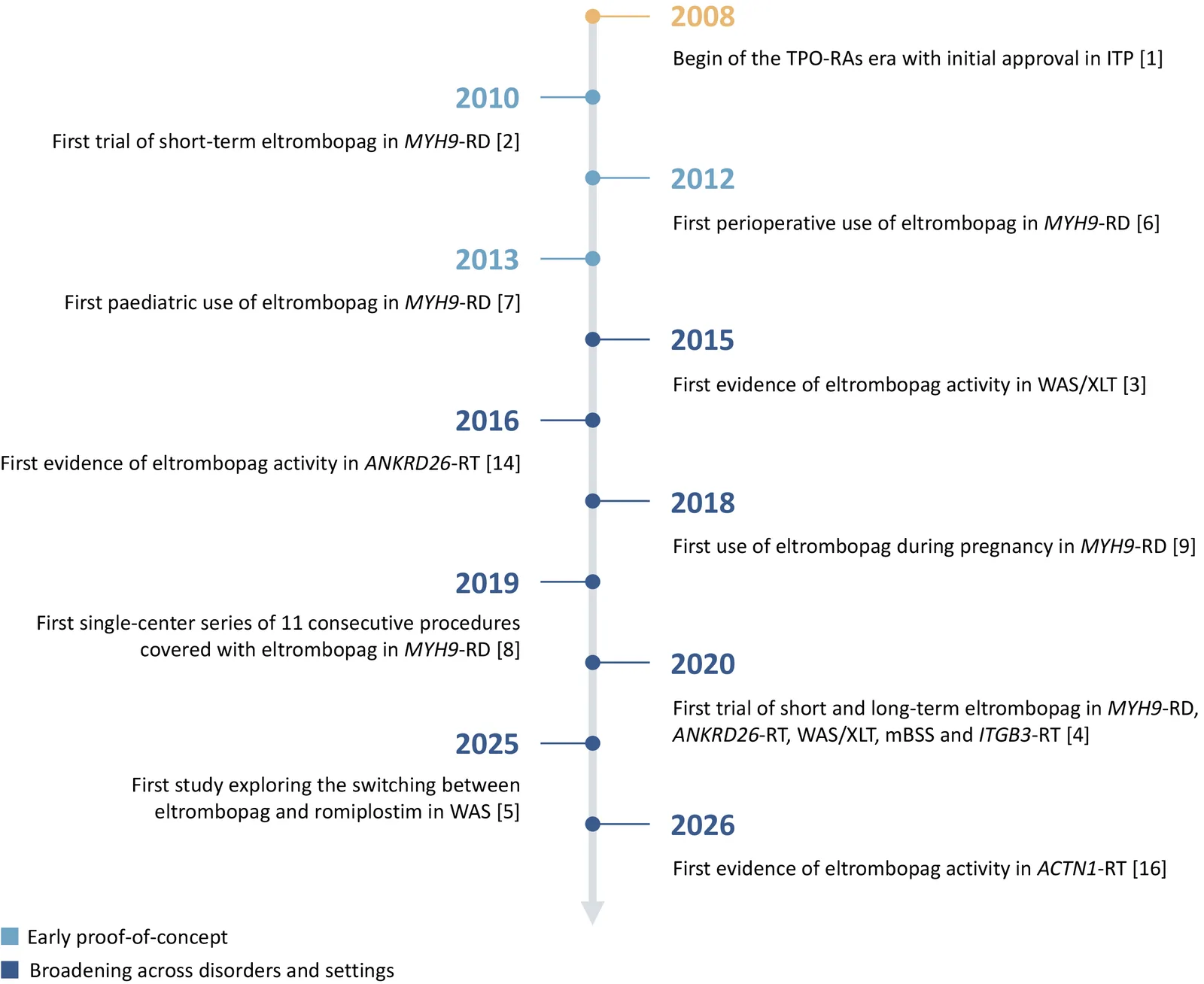
Chronological overview of key steps in the use of eltrombopag in inherited thrombocytopenias, from initial proof-of-concept in *MYH9*-related disease to expansion into additional disorders and perioperative settings. Data are derived from case series and small clinical trials. Numbers in parentheses refer to the references listed in the main text. *ACTN1*-RT, *ACTN1*-related thrombocytopenia; *ANKRD26*-RT, *ANKRD26*-related thrombocytopenia; *ITGB3*-RT, *ITGB3*-related thrombocytopenia; ITP, immune thrombocytopenia; mBSS, monoallelic Bernard–Soulier syndrome; *MYH9*-RD, *MYH9*-related disease; TPO-RA, thrombopoietin receptor agonist; WAS, Wiskott–Aldrich syndrome; XLT, X-linked thrombocytopenia.

In this issue of Research and Practice in Thrombosis and Haemostasis, de Oliveira et al. [16] build on this experience by systematically reporting short-term eltrombopag use in preparation for 15 elective procedures in 9 patients with *MYH9*-RD and 3 additional disorders: *ANKRD26*-RT, *ITGA2B*/*ITGB3*-related thrombocytopenia, and, for the first time, *ACTN1*-related thrombocytopenia. Platelet increases were achieved in 14 of 15 interventions, with a major response (platelet count > 100 × 10^9^/L or at least 2-fold higher than baseline) in about two-thirds of cases. Notably, no perioperative bleeding or need for platelet transfusion was reported, and only 1 nonresponder was observed, despite the inclusion of major orthopedic and cardiovascular surgeries.

These findings support short-term therapy with eltrombopag as a pragmatic alternative to platelet transfusion in appropriately selected patients with inherited thrombocytopenia undergoing elective surgery, reducing transfusion-related risks such as alloimmunization and subsequent platelet refractoriness [17].

From a mechanistic point of view, the inherited thrombocytopenias included in this series are characterized by impaired platelet formation with intact TPO-myeloproliferative leukemia protein signaling and largely preserved megakaryocyte maturation, a setting in which TPO-RAs are expected to be effective [18]. Although the need to increase a baseline platelet count of 68 × 10^9^/L before low-bleeding-risk venous surgery in 1 subject may be debatable, as this level is generally considered adequate for such procedures when platelet function is preserved, the responses observed in 2 *ACTN1*-RT patients provide the first clinical evidence of TPO-RA activity in this cytoskeletal disorder. By contrast, the lack of response in 1 of 2 patients with *ITGA2B/ITGB3*-related thrombocytopenia mirrors previous observations in this disorder and reaffirms that not all inherited thrombocytopenias, or all patients with the same defect, are equally responsive to eltrombopag [2,4]. However, a lack of response to 1 TPO-RA does not exclude a response to another. In fact, even in inherited thrombocytopenias, switching to an alternative agent may restore response in non- or partial responders to the first drug [5,19]. The favorable safety profile, with only 1 minor adverse event (headache) and no hepatotoxicity or thromboses, aligns with prior experience with short-term eltrombopag exposure in inherited thrombocytopenias [2,4,17].

From a good clinical practice perspective, the authors [16] adopted a pragmatic approach, initiating treatment sufficiently in advance of the scheduled intervention to allow possible dose escalation from 50 to 75 mg daily eltrombopag. In contrast, the absence of postoperative antithrombotic prophylaxis in any procedure reflects a bleeding-focused risk-benefit assessment and may indicate limited awareness that patients with inherited thrombocytopenias are not inherently protected against thrombosis [20]. One advantage of perioperative TPO-RAs is that they may enable the safe administration of standard antithrombotic prophylaxis, which should not be withheld merely because of inherited thrombocytopenia.

In genetic disorders, detailed molecular data are essential to confirm a precise diagnosis and explore genotype–phenotype correlations. While complying with local ethical regulations, the authors chose not to report the nucleotide and amino acid details of the identified variants. They provided references to previously reported mutations, along with information on variant type, the affected gene and protein domains, and the criteria used to assess pathogenicity for novel mutations. While this omission does not preclude interpretation of the reported findings, it remains a limitation. This issue, however, extends well beyond the present study [16] and reflects a broader, partly unresolved challenge in publishing rare genetic disorders. Scientific journals today must weigh not only the scientific validity of submitted contributions but also their compliance with regulations governing the handling of sensitive data, which may vary considerably across countries and contexts. Given the extreme rarity of some disorders, molecular data may themselves constitute identifying information, reducing protection for patient anonymity. Developing transparent modalities for controlled data sharing, without necessarily requiring full public disclosure, would therefore be highly valuable. In this context, establishing multicenter registries for inherited platelet disorders could foster constructive dialogue with local and supranational regulatory bodies in the search for balanced, ethically sound solutions.

Despite these limitations, the contribution by de Oliveira et al. [16] is timely and clinically relevant. It expands the cumulative perioperative experience with eltrombopag in inherited thrombocytopenias, introduces *ACTN1*-RT into the "responding" spectrum, and provides a practical, low-complexity approach that can be adopted outside specialized institutions.

Multicenter, prospective collaborations with standardized response criteria, harmonized perioperative platelet targets, and structured monitoring are now essential to transform this growing body of observational data into more robust clinical guidance. Particular attention should be paid to disorders such as *ANKRD26*-RT, given their recognized predisposition to hematological malignancies [4,14,17]. However, short-term perioperative use of TPO-RAs raises considerably fewer concerns about clonal evolution than prolonged exposure in a chronic treatment setting [17]. Such evidence could eventually open the door to official approval of TPO-RAs for selected inherited thrombocytopenias, although this goal remains challenging.

In the interim, this case series [16] reinforces a clear message: TPO-RAs should be considered a preferred option for elective surgical preparation in many patients with inherited thrombocytopenias. Their use requires adequate timing and careful risk-benefit evaluation, yet they already offer a pragmatic and effective alternative to platelet transfusion.

Evidence in rare disorders rarely arrives all at once but typically case by case, until a pattern becomes practice. The present series is one more important tile in that mosaic.
